# The genetics of water-use efficiency and its relation to growth in maritime pine

**DOI:** 10.1093/jxb/eru226

**Published:** 2014-07-01

**Authors:** Elisa Marguerit, Laurent Bouffier, Emilie Chancerel, Paolo Costa, Frédéric Lagane, Jean-Marc Guehl, Christophe Plomion, Oliver Brendel

**Affiliations:** ^1^University of Bordeaux, ISVV, EGFV, UMR 1287, F-33140 Villenave d’Ornon, France.; ^2^INRA, ISVV, EGFV, UMR 1287, F-33140 Villenave d’Ornon, France.; ^3^INRA, BIOGECO, UMR 1202, F-33610 Cestas, France.; ^4^University of Bordeaux, BIOGECO, UMR 1202, F-33400 Talence, France.; ^5^INRA, UMR 1137 ‘Ecologie et Ecophysiologie Forestières’, F-54280 Champenoux, France.; ^6^Université de Lorraine, UMR 1137 ‘Ecologie et Ecophysiologie Forestières’, Faculté des Sciences, F-54506 Vandoeuvre-les-Nancy, France.

**Keywords:** Breeding, carbon isotope composition, genotype×environment interaction, genetic variability, growth, heritability, maritime pine, QTL, water-use efficiency.

## Abstract

To meet the increasing demand of wood biomass worldwide in the context of climate change, developing improved forest tree varieties for high productivity in water-limited conditions is becoming a major issue. This involves breeding for genotypes combining high growth and moderate water loss and thus high water-use efficiency (WUE). The present work provides original data about the genetics of intrinsic WUE (the ratio between net CO_2_ assimilation rate and stomatal conductance, also estimated by carbon isotope composition of plant material; δ^13^C) and its relation to growth in *Pinus pinaster* Ait. First, heritability for δ^13^C was estimated (0.29) using a 15-year-old progeny trial (Landes provenance), with no significant differences among three sites contrasting in water availability. High intersite correlations (0.63–0.91) and significant but low genotype–environment interactions were detected. Secondly, the genetic architectures of δ^13^C and growth were studied in a three-generation inbred pedigree, introducing the genetic background of a more-drought-adapted parent (Corsican provenance), at ages of 2 years (greenhouse) and 9 years (plantation). One of the quantitative trait loci (QTLs) identified in the field experiment, explaining 67% of the phenotypic variance, was also found among the QTLs detected in the greenhouse experiment, where it colocalized with QTLs for intrinsic WUE and stomatal conductance. This work was able to show that higher WUE was not genetically linked to less growth, allowing thus genetic improvement of water use. As far as is known, the heritability and QTL effects estimated here are based on the highest number of genotypes measured to date.

## Introduction

From 2000 to 2010, the world lost 130 million hectares of forest, corresponding to 3.2% of the total forest area, but on the other hand, it has gained 78 million hectares, mainly as planted forests and natural forest expansion (Food and Agriculture Organization, 2012). This increase in planted forests corresponds to an increase in wood demand, mainly for fuel and industrial timbers, since natural forests cannot meet this demand anymore (Sutton, 1999)

Climate change will affect planted forests, through increased tree mortality (Allen *et al.*, 2010) and changes in net primary production (Hanson and Weltzin, 2000). While increasing temperature and rising atmospheric CO_2_ concentration are likely to increase growth (Way and Oren, 2010; Peñuelas *et al.*, 2011), a reduction in precipitation and a change of its distribution in the annual cycle will result in increased soil water deficits during the growing period and therefore in a reduced biomass production. Based on a modelling approach, Loustau *et al.* (2005) predicted a decrease of net primary production for *Pinus pinaster* forests in southwestern France because of a pronounced shift in seasonal rainfall from summer to winter. Dvorak (2012) argues that, considering the impact of plantation forestry on the hydrological cycle, the issue of water use will be one of the most important questions in many countries in the coming decades.

Two complementary strategies are being developed to adapt planted forests to these new climatic conditions: adaptive management of forest stands and adaptation of genetic material, either by planting new species which will use less water for biomass production and can better cope with a decrease of water availability or by adapting endogenous genetic resources by breeding. Existing forest tree breeding programmes mostly rely on improving wood biomass production and quality as well as disease and pest resistance (reviewed by Mullin *et al.*, 2011 for conifers). Adaptation to climate and soil characteristics is generally considered indirectly, by evaluating growth or survival. Given the challenges imposed by climatic changes, it becomes necessary that forest tree breeding also includes selection criteria related to the use of water.

To optimize the overall amount of water versus biomass accumulation, but also the maintenance of growth under reduced precipitation, is a crucial objective, which can be addressed by evaluating the ratio between biomass accumulation and water used (i.e. water-use efficiency, WUE). The direct estimation of WUE at the whole-tree level, for example by estimating time-integrated transpiration and biomass accumulation, is difficult (Kruse *et al.*, 2012), especially for adult trees, and it is impracticable to phenotype large populations of plants for estimations of genetic parameters. Screening of WUE of a large number of genotypes is commonly based on using a proxy, the carbon isotope composition (δ^13^C) of plant organic material, such as needles or wood (Brendel *et al.*, 2002, 2008; Dillen *et al.*, 2008; Gonzalez-Martinez *et al.*, 2008). Indeed, there is a linear positive relationship between carbon isotopic discrimination—a dimensionless measure of depletion in ^13^C in plant material as compared with atmospheric CO_2_ (carbon source for photosynthesis)—and intrinsic water-use efficiency (*W*_i_)—the ratio of net CO_2_ assimilation rate to stomatal conductance of water vapour (*A*/*g*_s_, Farquhar and Richards, 1984). For plant material grown in common environmental conditions, the δ^13^C of atmospheric CO_2_ is assumed common among plants and thus single measurements of δ^13^C of plant material can be used to estimate *W*_i_. *W*_i_ represents the leaf-level processes involved in whole-plant WUE, and screening for differences in *W*_i_ among tree genotypes is facilitated by the time- and/or crown-integrative nature of leaf or tree ring δ^13^C.

The main goal of the presented research was to study the feasibility of integrating WUE as a new selection criterion in the maritime pine breeding programme, similarly to the successful approach applied in breeding wheat cultivars in Australia (Richards, 2006). Maritime pine (*Pinus pinaster* Ait.) was used for several reasons. First, a breeding programme was launched in France in the 1960s to improve growth and straightness, and genetic resources are available to work both on genetic parameters and genetic architecture. Second, the planted forest in southwestern France, where this species spans over 1 million hectares, is expected to undergo reductions in water availability in the context of climate change, particularly during the growing season (Loustau *et al.*, 2005); water availability has a major impact on maritime pine growth (Aranda *et al.*, 2010; Corcuera *et al.*, 2010; Sanchez-Salguero *et al.*, 2012). Third, previous reports have shown that *W*_i_ (as estimated from δ^13^C) correlated well to WUE at the whole-plant level (Guehl *et al*., 1995, 1996) and displayed a large natural variability (Guehl *et al.*, 1995; Correia *et al.*, 2008), which might partly be due to differences in *W*_i_ among natural populations (Tognetti *et al.*, 2000). δ^13^C has also been shown to be heritable in this species, with low (Aranda *et al.*, 2010) to medium heritability (Brendel *et al.*, 2002; Lamy *et al.*, 2011), although it has been generally estimated with a rather low sample size and depends on the experimental set up and the genetic background. The genetic architecture of WUE has already been studied in one maritime pine family but, similarly to heritability, has also been estimated in a reduced genetic background and with a limited number of offspring (Brendel *et al.*, 2002).

In this context, the specific objectives of this work were two-fold: (i) to estimate genetic parameters for δ^13^C and growth, based on the Landes maritime pine breeding population, using a progeny trial established in three sites with contrasting soil water availability; and (ii) to compare the genetic architecture of δ^13^C and growth across different tree ages in a cross between a genotype from the breeding population and a genotype from a region with lower soil water availability. The rationale for the introduction of δ^13^C as new selection criteria for maritime pine breeding is discussed with the aim of providing improved varieties that maintain biomass production under water-shortage conditions.

## Materials and methods

### Plant material

Two types of plant material (Supplementary Table S1 available at *JXB* online) were used to study the genetics of WUE and its relation to growth: (i) a progeny trial from the Landes breeding population to estimate genetic parameters; and (ii) two F2 quantitative trait loci (QTLs) mapping populations, using the same grandparental genotypes, with one from the Landes breeding population and one from a Corsican population, for dissecting the genetic architecture of the studied traits.

### Progeny trial

In the 1960s, ‘plus’ trees were selected in the Landes de Gascogne forest (southwest of France) to constitute the base breeding population of maritime pine (G0 generation). This study considered a half-sib genetic trial (HST) consisting of progenies from G1 mother trees selected for growth and stem straightness and crossed with a polymix (mix of pollen from 42 G1 trees). The HST was established in three sites (Escource, Lagnereau, and Cestas) with a density of 1250 trees ha^–1^ (4×2 m). A total of 196 (Escource), 156 (Lagnereau) and 174 (Cestas) half-sib families were planted in each site, in 35 randomized complete blocks with a single-tree plot design. Seedlings were sown in 1995 and raised for 1 year in a nursery before being transferred to the field. These sites are characterized by a sandy podzol. Following Augusto *et al.* (2010), despite similar precipitations across the three sites, Escource is classified as a dry heathland, whereas Lagnereau and Cestas can be considered as semihumid and humid heathlands, respectively. These three sites correspond to those defined by Jolivet *et al.* (2007) based on understorey composition and depth of the water table: dry heathland (understorey vegetation dominated by *Erica cinerea* and *Calluna vulgaris*, deep water table), mesophylous (semihumid) heathland (*Pteridium aquilinum*, moderate depth of the water table), wet (humid) heathland (*Molinia caerulea*, water table close to the topsoil). There are also differences in soil nutrients, with less nitrogen and carbon contents at Escource compared with the other two sites (Supplementary Table S2 available at *JXB* online).

From this progeny trial 50 half-sib families were chosen, present in all three sites and representing the diversity in growth observed at age 12 in the G1 population (Supplementary Fig. S1 available at *JXB* online). Between 10 and 13 trees per half-sib family and per site were phenotyped for WUE (i.e. in total 634 trees at Escource, 636 trees at Lagnereau, and 631 trees at Cestas).

### QTL mapping populations

A three-generation inbred mapping population (F2) was used to study the genetic architecture of quantitative traits. To generate the F2 population, selfed seeds were obtained from a single hybrid tree (accession H12) derived from a controlled cross between a Landes genotype (accession L146, from Arengosse, used as female) and a Corsican genotype (accession C10, from Corte, used as male). The closest meteorological stations (Meteo-France) were used to describe the climate for the origins of the parental trees: Sabres for the Landes genotype and Corte for the Corsican genotype. Based on data from 1995 to 2010, Sabres showed significantly higher annual precipitation (1108mm) compared with Corte (818mm), whereas monthly mean potential evapotranspiration (PET) was significantly higher in Corte (80mm) compared with Sabres (68mm), especially during the growth period (May to August). The whole mapping population was divided into two seed lots. The first population (F2 greenhouse) consisted of 200 genotypes raised in 1998 in 4-l pots in a greenhouse and were subjected in their second year to a drying cycle by withholding watering for 6 weeks, after a last watering to field capacity. The second population (F2 plantation) consisted of 477 genotypes sown in 1998 and raised for 1 year in a nursery before being transferred to the field. This trial was located at Lacanau de Mios (Gironde, France), a semihumid heathland, with a density of 1250 trees ha^–1^ (4 m×2 m) and trees were 9 years old at the time of phenotyping (2008).

### Phenotyping

#### Traits related to WUE 

For the HST and the plantation populations, δ^13^C was measured on a set of three annual rings (2006, 2007, and 2008), which were extracted from increment cores. Extractives were removed by soaking the cores in pentane for 24h. Wood was cut by hand into small pieces, preground in a centrifugal mill (Tecator, Cyclotech 1093, Sample Mill, Höganäs, Sweden) and milled to a fine powder in a ball mill (MM2000, Retsch, Haan, Germany). For the greenhouse population, δ^13^C was measured on bulk needle dry matter. Four pairs of needles were harvested from the upper third of the main stem for 200 genotypes before the drying cycle (δ^13^C_WW_stem) and after the drying cycle (δ^13^C_D_stem) for 189 genotypes. Four pairs of needles were also sampled from a lateral shoot for 107 genotypes before the drying cycle (δ^13^C_WW_shoot) and after the drying cycle (δ^13^C_D_shoot) for 105 genotypes. The needles were ground to a fine powder using a ball mill. For all analyses, a 1mg subsample of the plant material (wood or needle dry matter) was combusted in an elemental analyser (Carlo Erba NA1500, Milano, Italy). δ^13^C was determined with a coupled continuous-flow isotope ratio mass spectrometer (Delta S, Finnigan MAT, Bremen, Germany). Carbon isotope composition (δ^13^C expressed in ‰) was calculated relative to the VPDB standard as (Craig, 1957):

$$\delta^{13}C=\frac{R_{sa}-R_{sd}}{R_{sd}}\times 1000$$

where *R*_*sa*_ and *R*_*sd*_ are the ^13^C/^12^C ratios of the sample and the standard, respectively.

For the greenhouse population, *W*_i_ was also evaluated directly from the ratio of net assimilation rate (*A*, μmol m^–2^ s^–1^) to stomatal conductance of water vapour (*g*_s_, mmol m^–2^ s^–1^). *A* and *g*_s_ were assessed once before the drying cycle using a portable gas-exchange system with a cylindrical chamber (CIRAS-1 with ‘pod chamber’, PP-Systems, Herts, UK) on one pair of 1-year-old needles per plant at a chamber CO_2_ concentration of 1200 µmol mol^–1^ and a saturating photosynthetic photon flux density (1500 μmol m^–2^ s^–1^). The elevated CO_2_ concentration during gas-exchange measurement does not affect *g*_s_ in maritime pine compared with an ambient CO_2_ concentration (Picon *et al.*, 1996) and was used here to allow an assessment of *A* under nonlimiting CO_2_ concentration. The temperature in the measurement chamber ranged from 20 to 28 °C. Needle area was estimated from the length and the width of the needle included in the chamber, assuming a half-cylindrical needle shape.

#### Traits related to growth 

Increment cores were sampled in the HST and the plantation populations. Rings were indexed by calendar year and ring width was evaluated using an indirect-reading X-ray densitometer (Seifert Isovolt 3003, GE Inspection Technologies, France) as described by Bouffier *et al.* (2008). In order to analyse comparable sets of three rings for δ^13^C and growth, this work considered ring width means for years 2006–2008 for the HST and plantation populations, corresponding to cambial age 10–12 years and 7–9 years, respectively.

Total height and circumference at breast height were also measured in 2008 at age 12 in the three sites of the HST. Woody biomass was evaluated in the greenhouse population based on weight of roots and primary and secondary axes without needles.

### Linkage map construction and comparative QTL mapping

For the greenhouse population, a linkage map (here referred as MapF2g) was built from DNA of 200 haploid megagametophytes with 235 amplified fragment length polymorphisms (AFLPs), 127 random amplified polymorphic DNA (RAPDs), and 32 protein markers resulting in 13 linkage groups (LGs, LG 2 was split in two subgroups) (Costa *et al.*, 2000). The average distance between two framework loci was 15 cM (Kosambi). In this study, MapF2g was used in order to detect QTLs for δ^13^C, *A*, *g*_s_, and *A*/*g*_s_.

For the plantation population, a linkage map (MapF2p) was established from the genotyping of 477 offspring with 248 single-nucleotide polymorphism (SNP) markers (C Plomion, E Chancerel, I Lesur, data unpublished), resulting in 12 LGs corresponding to the haploid number of chromosomes of the pine genome. The length of the map was 1754 cM (146 cM per LG on average) with a density of one SNP every 7 cM. In this study, MapF2p was used to detect QTLs for δ^13^C and ring width.

In order to compare QTLs detected for the greenhouse and plantation populations with QTLs detected by Brendel *et al.* (2002), both linkage maps (MapF2g and MapF2p) were aligned with two other already published linkage maps (Map1 and Map2). Map1 was developed in the mid 1990s with another previous set of haploid megagametophytes from the same three-generation inbred mapping population using 436 RAPD markers (Plomion *et al.*, 1995) and was later completed with five single-strand conformation polymorphisms (Plomion *et al.*, 1999) and 17 protein markers (Plomion *et al.*, 1997). Map1 and MapF2g were aligned by Costa *et al.* (2000) based on a set of 40 common markers (seven proteins and 33 RAPDs). Map2 was developed by Chagné *et al*. (2002) using 620 AFLP markers genotyped on a subset of 90 full-sib individuals (Map2♀ and Map2♂). The genotyping was extended to the whole progeny (186 full-sibs), using 219 evenly spaced AFLP markers segregating 1:1 (116 for the female map and 103 for the male map) as described by Pot *et al.* (2006). These two parental maps were used for QTL detection for δ^13^C and growth by Brendel *et al.* (2002). Map1 and Map2 were also aligned using 24 common markers (Single Sequence Repeats (SSRs) and Express Sequence Tag Polymorphisms (EST-Ps)) developed by Plomion *et al.* (1999), Chagné *et al*. (2003, 2004), and Guevara *et al.* (2005). Map2 and MapF2p were finally aligned by Chancerel *et al.* (2011) with 98 SNP markers. Therefore, homologous LGs and orientations could be unambiguously identified between the four maps to compare the QTLs here (MapF2g and MapF2p) with those of Brendel *et al.* (2002) (Map2). A description of the linkage maps is available in Supplementary Table S3 available at *JXB* online.

### Statistical analyses

Analysis of phenotypic correlations among traits was carried out using linear regression analysis.

#### Estimation of genetic parameters 

Genetic parameters were first estimated from the HST within each site (model 1) using the following linear mixed model (interactions between block and family effects were not significant) for univariate or bivariate analyses:

y=Xb+Zf+e

where *y* is the vector of phenotypic individual observations, *b* is the vector of fixed block effects, *f* is the vector of random family effects, *e* is the residual, and *X* and *Z* are the incidence matrixes linking phenotypic observations to fixed and random effects.

Variances of random effects were defined as follows:

$$Var\left(f\right)=G=Id.\sigma_{f}^{2}\text{ and }Var\left(e\right)=R=Id.\sigma_{e}^{2}.$$

Phenotypic ($\sigma_{P}^{2}$) and additive genetic ($\sigma_{A}^{2}$) variances were obtained from the following equations:

$$\sigma_{P}^{2}=\sigma_{f}^{2}+\sigma_{e}^{2}\;\;\text{and }\sigma_{A}^{2}=4\times \sigma_{f}^{2}$$

because the families were assumed to be half-sibs.

Genetic and phenotypic coefficients of variation (CV_A_ and CV_P_) were derived from these variances:

$$CV_{A}=\frac{\sigma_{A}(X)}{\overset{ˉ}{X}}\text{ and }CV_{P}=\frac{\sigma_{P(X)}}{\overset{ˉ}{X}}$$

where $\overset{ˉ}{X}$ is the mean of the studied trait. As δ^13^C is a relative measurement, its coefficient of variation was only considered for intersite and not for intertrait comparisons (Brendel, 2014).

Narrow-sense heritability (*h* ^*2*^) was estimated as:

$$h^{2}=\frac{\sigma_{A}^{2}}{\sigma_{P}^{2}}.$$

The phenotypic (*r*_*P*_) and additive genetic (*r*_*A*_) correlations between two traits, *y*_1_ and *y*_2_, were estimated as follows:

$$r_{P}=\frac{Cov_{P}\left(y_{1},y_{2}\right)}{\sqrt{\sigma_{Py_{1}}^{2}\times \sigma_{Py_{2}}^{2}}}\text{ and }r_{A}=\frac{Cov_{A}\left(y_{1},y_{2}\right)}{\sqrt{\sigma_{Ay_{1}}^{2}\times \sigma_{Ay_{2}}^{2}}}.$$

Intersite correlations were analysed from the HST, combining data from the three sites (Escource, Lagnereau, and Cestas) in a similar linear mixed model as described previously, with specific family covariances fitted between each site (model 2). G and R matrices (the variance - covariance matrix of families’ effect (ie. genetic effect) and the variance - covariance matrix of the residual effect, respectively) can then be described as follows:

$$\begin{array}{l} G=\left(\begin{array}{ccc} \sigma_{f_{Escource}}^{2} & \sigma_{f_{Lagnereau,Escource}} & \sigma_{f_{Cestas,Escource}}\\ \sigma_{f_{Escource,Lagnereau}} & \sigma_{f_{Lagnereau}}^{2} & \sigma_{f_{Cestas,Lagnereau}}\\ \sigma_{f_{Escource,Cestas}} & \sigma_{f_{Lagnereau,Cestas}} & \sigma_{f_{Cestas}}^{2} \end{array}\right)⊗I_{f}\text{ with }I_{f}\text{being the }\\ \text{ number of families},\text{ and} \end{array}$$

$$\begin{array}{l} R=\sigma_{e_{Escource}}^{2}.I_{Escource}⊕\sigma_{e_{Lagnereau}}^{2}.I_{Lagnereau}⊕\sigma_{e_{Cestas}}^{2}.I_{Cestas}\;\text{with }\\ \;I_{site}\text{being the number of individuals per site}. \end{array}$$

Intersite genetic correlations were estimated as:

$$\frac{\sigma_{f_{site_{i},site_{j}}}}{\sqrt{\sigma_{fsite_{i}}^{2}\times \sigma_{fsite_{j}}^{2}}}.$$

Analyses were conducted with the restricted maximum-likelihood method using the software ASReml (Gilmour *et al.*, 2009) and the standard errors of the estimates were calculated using ASReml with a standard Taylor series approximation (Gilmour *et al.*, 2009). Statistical models were compared by likelihood ratio tests.

The expected response (R) after a selection step can be expressed as (Lynch and Walsh, 1998):

$$\text{R}=i\times \sigma_{P}\times h^{2}$$

where *i*=2.67 (selection of the 1% best individuals), σ_P_ is phenotypic standard deviation, and *h* ^*2*^ is individual heritability.

#### QTL mapping 

δ^13^C, gas-exchange data (*A*, *g*_s_, *A*/*g*_s_), ring width, and biomass were analysed. Data were not transformed despite slight deviations from normality because the interval mapping method is robust against this assumption (Rebaï, 1997). Moreover, permutation analysis provides valid thresholds for nonstandard situations such as nonnormal distributions (Members of the Complex Trait Consortium, 2003). QTL detection was conducted using MultiQTL version 2.6 (Haifa 2005, http://www.multiqtl.com) as described by Marguerit *et al.* (2012).

As the maps of the hybrid tree were constructed using either megagametophytes (MapF2g) or needle DNA (MapF2p), QTL detection was carried out under a back-cross model and a classical F2 model, respectively.

For the greenhouse population, a multi-environment analysis was also carried out for δ^13^C estimated from the two needle positions (stem or shoot) and sampling times before and after the drying cycle. As no needle growth had taken place during the drought stress and as there were strong positive correlations between δ^13^C_WW_stem, δ^13^C_D_stem, δ^13^C_WW_shoot, and δ^13^C_D_shoot, with slopes close to 1 (Supplementary Table S4 available at *JXB* online), these data were used as technical replicates in order to increase the statistical power of the QTL analysis. By reducing environmental variance, this method enhances the detection power of low-effect QTLs (Jansen *et al.*, 1995).

## Results

### Variation in δ^13^C and growth

**Fig. 1.**
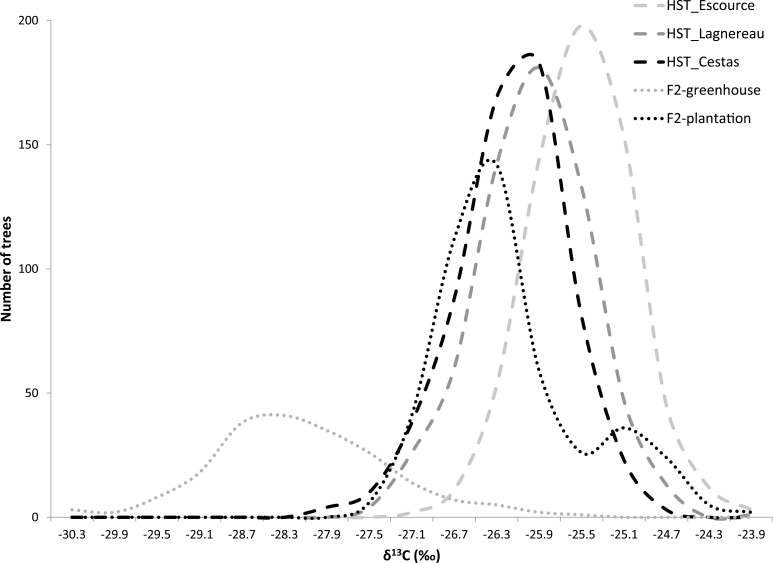
Distribution of δ^13^C in the HST populations (Escource, Lagnereau, Cestas) and the F2 greenhouse and plantation populations.

At 12 years (2008), trees on the three sites showed significant differences for growth (means, 874cm at Escource, 965cm at Lagnereau, and 1043cm at Cestas). The distribution of δ^13^C for the HST populations (three sites) and the greenhouse and plantation populations are presented in Fig. 1 and Supplementary Table S5 available at *JXB* online. δ^13^C was normally distributed except for the plantation population (Shapiro–Wilkinson test). In the HST populations, δ^13^C was higher at Escource (dry site) compared to the two other sites, but no differences were found in CV_P_. As expected due to similar experimental conditions (field experiments with identical tree density, similar climate, and sandy podzol), δ^13^C was comparable for the HST populations (mean of the three sites –26.1‰) and the plantation population (–26.4‰) but plants of the greenhouse population showed clearly lower δ^13^C (–28.3‰) which may be attributed to the lower δ^13^C of the atmospheric CO_2_ in the greenhouse (mixing of outside CO_2_ with ^13^C-depleted CO_2_ issued from plant and soil respiration). Both mapping populations clearly exhibited higher CV_P_ for δ^13^C than the HST populations. The observed variance was higher in the greenhouse population compared with the plantation population (3.1 versus 2.6%) and the greenhouse population showed a clear bimodal distribution with a peak ratio of 4:1.

### Genetic parameter estimations for traits related to WUE and growth

δ^13^C exhibited significant additive genetic variability in the three HST sites (Supplementary Table S5 available at *JXB* online). Heritability of δ^13^C was first estimated within each site (model 1): 0.23 at Escource, 0.41 at Lagnereau, and 0.34 at Cestas. These estimates (Table 1) were higher than heritabilities obtained for integrated growth traits (circumference and height at age 12) but similar to heritability of growth (ring width 2006–2008) evaluated on the same annual rings as used for δ^13^C. Because additive genetic variability for δ^13^C and ring width were not significantly different among sites, overall heritabilities of (mean±SE) 0.29±0.07 or 0.25±0.07, respectively, could be estimated (model 2). Based on these genetic parameters, if the best 1% of individuals were selected from the HST for δ^13^C, the expected response would be a shift of 0.4‰ for the δ^13^C population mean.

**Table 1.** Heritability for δ^13^C, circumference and height at age 12 years, and mean ring width from 2006 to 2008 estimated in the half-sib genetic trial populationsValues are mean±standard error.

	Escource	Lagnereau	Cestas
δ^13^C	0.23±0.11	0.41±0.14	0.34±0.13
Circumference at 12 years	0.06±0.08	0.17±0.10	0.27±0.12
Height at 12 years	0.07±0.08	0.32±0.12	0.08±0.09
Ring width 2006–2008	0.31±0.12	0.27±0.12	0.42±0.14

The intersite genetic correlations estimated for δ^13^C between Escource, Lagnereau, and Cestas with model 2 were high (Table 2): from 0.63 between Escource and Cestas to 0.91 between Lagnereau and Cestas. The correlations between δ^13^C and growth-related traits estimated within each site (Table 3) were either not significant or positive. No significant genetic correlation was found between δ^13^C and circumference at age 12 whatever the site considered, but genetic correlations were significant between δ^13^C and height at age 12 at Escource (0.30, *P*<0.05) and Lagnereau (0.32, *P*<0.05). A highly significant positive correlation between δ^13^C and ring width (0.40, *P*<0.01) was also obtained at Escource.

**Table 2.** Across-site correlations for δ^13^C and ring width 2006–2008 estimated in the half-sib genetic trial populationsGenetic correlations for δ^13^C are above the diagonal; ring width 2006–2008 are below the diagonal. All correlations are significantly different from 0.

	Escource	Lagnereau	Cestas
Escource		0.89	0.63
Lagnereau	0.44		0.91
Cestas	0.76	0.70	

**Table 3.** Phenotypic and genetic correlations between δ^13^C and growth-related traits in the half-sib genetic trial populations

	Phenotypic correlation			Genetic correlation		
	Escource	Lagnereau	Cestas	Escource	Lagnereau	Cestas
Circumference at 12 years	0.31*	0.15	0.04	0.26	0.21	–0.02
Height at 12 years	0.31*	0.27*	0.08	0.30*	0.32*	0.08
Ring width 2006–2008	0.44**	0.18	0.31*	0.40**	0.19	0.29*

*P<0.05; **P<0.01.

### QTL mapping for traits related to WUE and growth

In addition to the large variation observed for δ^13^C in the greenhouse population, a large variation for *W*_i_ was also observed (CV_P_=31%). This variation was mainly determined by the variation of stomatal conductance (*g*_s_), which was shown by its equally high CV_P_ of 31%, compared to the lower CV_P_ for assimilation rate (*A*) of 14%. *A* and *g*_s_ were significantly correlated (*R*=0.42, *P*<0.01), with a low correlation coefficient and a low slope (0.06 μmol mmol^–1^; Supplementary Table S6 and Supplementary Fig. S2 available at *JXB* online). Further, the correlation between *W*_i_ and δ^13^C was significant, with a slope of 0.03, indicating a variation of 0.3 μmol mmol^–1^ per ‰ change. Correlation between δ^13^C_WW_shoot and *A* was negative and lower (*R*=–0.19, *P*<0.05) than that between δ^13^C_WW_shoot and *g*_s_ (*R*=–0.53, *P*<0.01). The correlation between biomass and *g*_s_ was higher (*R*=–0.27, *P*<0.01) than with *A* (*R*=–0.07, not significant).

**Fig. 2.**
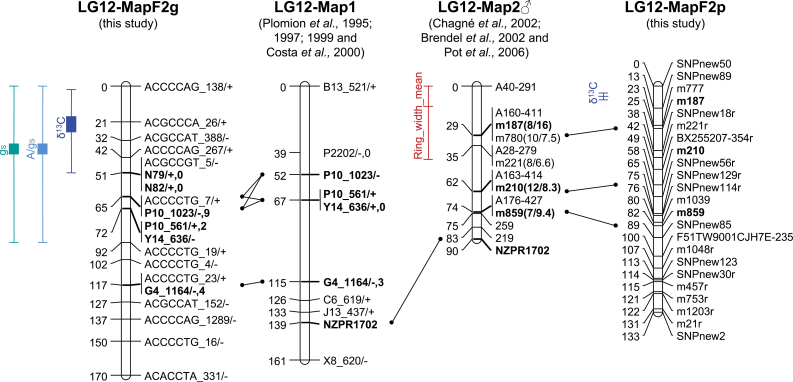
Linkage group 12 (LG12) for the four genetic linkage maps of maritime pine and associated QTLs. MapF2g corresponds to the genetic map published by Costa *et al.* (2000) from the F2 greenhouse population; Map1 corresponds to the genetic map published by Plomion *et al.* (1995) and used as bridge to align MapF2g to Map2♂; Map2♂ corresponds to the male map published by Chagné *et al.* (2002) on which SNP markers were added as accessory markers by Chancerel *et al.* (2011). The framework Map2 was used for QTL detection of water-use efficiency by Brendel *et al.* (2002); and MapF2p corresponds to the map established by Chancerel *et al.* (2013) from the analysis of the F2 plantation population. Bars indicate the range of mean positions from *L* and *L*_*BS*_ (see Tables 4, 5 and Supplemental Table S7 available at *JXB* online). Whiskers are the 95% confidence interval around *L*_*BS*_ (this figure is available in colour at *JXB* online).

For the greenhouse population, the multi-environment QTL analysis identified nine QTLs for δ^13^C (Table 4). The detected single-environment QTLs explained from 31% to 59% of the phenotypic explained variance (PEV), depending on environment (needle position and water status). The strongest QTL was located on LG 12, explaining 6–10% of PEV, with a QTL effect average of 0.5‰. This QTL was the only one detected when using a single-environment QTL model for each of the δ^13^C measurements (data not shown). One QTL was also detected for the *A*/*g*_s_ ratio (*W*_i_) and *g*_s_, whereas none was identified for *A* (Supplementary Table S7 available at *JXB* online). The QTLs for *A*/*g*_s_ and *g*_s_ were both located in the same region on LG 12, close to the QTL for δ^13^C (Fig. 2) and explained more than 6% of PEV.

**Table 4.** Significant quantitative trait loci detected by the multienvironment analysis for water-use efficiency estimated by δ^13^C for the F2 greenhouse population (200 plantlets)Because the linkage map of the hybrid tree of the F2 mapping population was generated using haploid mega-gametophyte DNA extracted from each F2 plant (i.e. maternal contribution only), the QTL effect corresponds to an allelic substitution (allele a versus A). LG, linkage group; *P*_*G*_, level of significance at the genomic level; *L*, position of QTL on the LG estimated from original data-set; *L*_*BS*_, position of QTL as calculated from bootstrap analysis (mean±standard deviation); WW, well-watered conditions; D, drought. Asterisks indicate significant differences from zero.

LG	LOD	*P*_G_	*L* (cM)	*L*_*BS*_ (cM)	95% CI	Percentage of explained variance (%)				Allelic substitution effect			
						WW_stem (31%)	WW_shoot (58%)	D_stem (32%)	D_shoot (59%)	WW_ stem	WW_ shoot	D_stem	D_shoot
1	5.2	0.027	164.2	154.4±33.2	89.3–176.6	0.7	2.8	2.6	3.2	–0.14	–0.33*	–0.26*	–0.30*
2b	13.3	0.0009	34.1	35.3±4.8	25.8–44.8	3.6	9.3	3.6	11.9	0.34*	0.59*	0.30*	0.58*
3	6.0	0.027	18.5	26.4±28.9	0–83.0	1.2	3.8	1.7	4.2	–0.19	–0.38	–0.21	–0.35
4	9.6	0.0006	134.4	133.8±5.7	122.7–144.9	4.5	6.1	4.3	2.5	–0.38*	–0.48*	–0.33*	–0.27*
5	4.8	0.044	43.3	49.6±26.6	0–101.7	0.6	4.7	2.0	8.9	0.14	0.42	0.22	0.50
9	8.0	0.0009	81.6	83.0±6.4	70.5–95.4	3.7	6.5	4.2	1.9	–0.34*	–0.50*	–0.33*	–0.23
10	10.5	0.0005	181.0	179.7±3.8	172.3–187.0	2.0	7.4	2.3	9.0	0.25*	0.53*	0.24*	0.50*
11	8.9	0.0006	4.0	4.7±5.1	0–14.6	3.8	8.9	1.7	5.9	–0.34*	–0.58*	–0.21	–0.41*
12	14.3	0.0004	17.6	26.5±12.5	1.9–51.1	8.5	5.6	6.3	10.4	0.52*	0.46*	0.40*	0.54*

**Table 5.** Significant quantitative trait loci of water-use efficiency estimated by δ^13^C and ring width mean for the F2 plantation population (trees)*LG*, linkage group; *P*_*G*_, level of significance at the genomic level; *L*, position of QTL on the LG estimated from original data-set; *L*_*BS*_, position of QTL as calculated from bootstrap analysis (mean±standard deviation); PEV, percentage of explained phenotypic variance; PEV_a_, percentage of explained additive variance; *a*, additive allelic effect; *d*, dominance allelic effect. Asterisks indicate significant differences from zero.

Trait	*n*	LG	LOD	*P*_G_	*L* (cM)	*L*_*BS*_ (cM)	95% CI	PEV (%)	PEV_ad_ (%)	*a*	*d*
δ^13^C	447	8	4.4	0.013	60.0	47.9±20.7	7.2–88.5	1.6	0.1	–0.04	–0.18
		12	81.5	0.0005	6.0	6.1±0.9	4.4–7.8	66.7	46.3	1.38*	–0.65*
Ring width mean	460	1	5.2	0.0008	142.2	137.1±20.9	96.1–144.8	4.9	3.3	–0.81*	0.39*
		10	6.9	0.0006	12.9	23.7±24.3	0–71.3	6.1	5.6	1.05*	0.22

For the plantation population, the QTL analysis identified one large-effect QTL on LG 12, explaining 66.7% of PEV, as well as a minor QTL on LG 8. The QTL on LG 12 presented positive additive and dominant effects (Table 5). Its confidence interval overlapped with those of the QTLs detected for δ^13^C, *A*/*g*_s_, and *g*_s_ in the greenhouse population (Table 4 and Supplementary Table S7 available at *JXB* online). Working with a genetic map of the F2 population using codominant markers allowed to study the contribution of alleles at the two flanking SNP markers of this QTL. Homozygous genotypes for the Landes allele as well as the heterozygous genotypes presented a significant lower δ^13^C compared with the homozygous genotypes for the Corsican allele (Supplementary Table S8 available at *JXB* online). The differences between genotypes with one or two copies of the Landes allele versus the homozygous genotypes for the Corsican allele (i.e. no dose of Landes allele) was around 1‰. The interaction of these alleles showed that the Corsican allele was recessive.

The phenotypic correlation between δ^13^C and ring width mean was not significant for the complete plantation population (*R*=–0.04) as well as when F2 offsprings of the three genotypes (homozygous Landes and Corsican, heterozygous) were analysed separately (−0.01<R<–0.05).

Two QTLs were detected for ring width mean, explaining together 11% of PEV. None of them was located within the confidence interval of the δ^13^C QTL (Table 5).

## Discussion

### Limitations

Quantitative genetic studies in trees are subject to the following limitations, which could explain the reduced heritability and the detection of only a few QTLs in this study.

With respect to genetic parameter estimation, the large size of the HST (>5 ha for each site) implies a large uncontrolled heterogeneity of the soil. This can have an effect on the phenotype and thus complicate the estimation of genetic parameters. However, the large families used in this study made it possible to establish trials consisting of blocks, which can be used to take this heterogeneity into account.

Many tree species cannot be easily propagated vegetatively (including maritime pine), therefore QTL studies in these species are based on a single-tree plot design without ‘clonal’ replicates. Consequently, environmental heterogeneity, especially of the soil, cannot be controlled by introducing blocks into the experimental design. Spatial variation of soil properties could therefore have an impact on the estimation of the phenotype and thus result in less statistical power to detect low-effect QTLs. Therefore, before the data analysis, this work verified for the QTL field experiment, using a spatial analysis of the phenotype of the measured trees, that no spatial pattern was detectable (Supplementary Fig. S3 available at *JXB* online).

Traits of interest (growth, wood properties, resistance to biotic and abiotic stresses) often represent an integration over several growing seasons, which will average out annual environmental variation when the genotype×environment interaction is not particularly strong and thus result in a more robust estimation of phenotypic values for each genotype.

### Genetic parameters of the breeding population

Genetic parameters were estimated in the Landes provenance as this genetic material constitutes the main French maritime pine breeding population. δ^13^C heritability measured across different environments was moderate (0.29±0.07) and did not vary significantly among sites. It was similar to that found for ring width growth (0.25±0.07) evaluated for the same years but slightly higher than for total height and circumference at the age of 12. The genetic parameters of δ^13^C were accurately estimated in this study due to the presence of a large genetic variability in the G1 breeding population on the one hand and the analysis of the same genetic material in three sites contrasting in water availability on the other. Thus, this robust estimation of δ^13^C heritability was obtained using a wider genetic background within the breeding population compared to the first estimation of 0.17±0.06 provided by Brendel *et al.* (2002) using a 12×12 half-diallel (i.e. based on 12 genotypes). In the current data set of more than 1800 trees, the genetic variability for δ^13^C was as large as the one found across the natural range of the species (Guehl *et al.*, 1996). Despite the different soil conditions, the genetic within-site variability was not different among sites, suggesting only a few genotype×environment interactions.

In other coniferous species, the highest narrow-sense heritabilities estimated for δ^13^C were reported by Prasolova *et al.* (2001) for *Araucaria cunninghamii* (0.66–0.72) and by Johnsen *et al.* (1999) for *Picea mariana* (0.54), whereas very low values (<0.1) were observed for *Pinus taeda* (Baltunis *et al.*, 2008). These contrasting estimates illustrate the complex determinism of δ^13^C and its underlying leaf-level processes which are under both genetic and environmental control. On a more technical side, this variability in heritability estimates can also result from poor experimental designs in terms of sample size: 22 and 26 open-pollinated families with 101 and 130 trees for Prasolova *et al.* (2001); 18 full-sib families (from seven parents only) with 820 trees for Johnsen *et al.* (1999); 61 full-sib families (from 32 parents) with 1027 trees for Baltunis *et al.* (2008).

Intersite correlations for δ^13^C revealed significant but low genotype×environment interactions, which suggest that the ranking of families was conserved among sites. Sites that were environmentally closer showed a stronger correlation, suggesting that genotype–environment interactions might become stronger when more contrasting planting environments are considered. Moreover, mostly the intersite correlations for δ^13^C were higher than those obtained for growth traits, indicating stronger genotype×environment interactions for the latter. This finding is interesting from a breeding perspective, as selection for higher WUE might be more stable than selection for growth.

### Genetic architecture of δ^13^C and *W*_i_ in a Landes × Corsican genetic background

The genetic architecture of δ^13^C has been previously studied within the Landes provenance genetic background (Brendel *et al.*, 2002) and one strong QTL for δ^13^C was detected. Here, a wider genetic background, a Landes × Corsica F2 mapping pedigree, was used for QTL detection for WUE- and growth-related traits in two independent experimental settings: a greenhouse with 2-year-old seedlings and a field plantation with 9-year-old trees.

In the plantation population, a major QTL region linked to δ^13^C on LG 12 was detected (accounting for 60% of PEV) that was also detected in the greenhouse population. This QTL had not been detected in the intra-Landes pedigree used by Brendel *et al.* (2002), and vice versa, the major QTL detected by Brendel *et al.* (2002) was not detected in this study (Supplementary Table S9 available at *JXB* online). This suggests that the underlying genetic factors are different between the pedigrees. Compared to the geographical origin of the Landes parent, the original population of the Corsican parent is subjected to higher annual potential evapotranspiration, especially in the summer. This results probably in a higher summer water deficit, which might have caused specific strategies (therefore different genetic mechanisms) to cope with soil water deficit.

The greenhouse experiment allowed a more detailed study of traits related to WUE. Here, the alignment of the different LGs (Fig. 2) allowed this work to show that the major QTL for δ^13^C located on LG 12 in the plantation population: (i) colocalized with the QTL detected in the greenhouse population; and (ii) colocalized with a QTL for a direct measurement of *W*_i_ and with a QTL for *g*_s_. Similarly to the Brendel *et al.* (2008) study on *Quercus robur* L., the colocalization of the QTL as well as the positive phenotypic correlation between δ^13^C and *A*/*g*_s_ confirmed the major QTL for δ^13^C as a QTL for WUE rather than for one of the Farquhar *et al.*, (1989) model parameters, which depend on biology (e.g. day respiration, photorespiration, mesophyll conductance of CO_2_). Further, the colocalization with a QTL for *g*_s_ suggests an introduction of alleles by the Corsican, supposedly more drought adapted parent, which might be related to stomatal responses, resulting in genotypes that are more efficient in water use. The correlations between gas exchange related traits and δ^13^C suggest that the variability of WUE in this cross was probably driven by variation in *g*_s_ rather than *A*. The coefficients of variations of *A* and *g*_s_ suggest a larger diversity of the latter in the greenhouse population. Additionally, the significant correlation between the two traits had a low slope, suggesting only a relatively low impact of *g*_s_ on *A*. Thus, within this family, a lower *g*_s_ effectively reduced water consumption, increasing WUE with only a low impact on aboveground biomass growth. It is worth noting that the impact of this genomic region on LG 12, which controlled the variability of δ^13^C, became much stronger in the field plantation (8–67%), even though these trees were much older than the seedlings in the greenhouse, and measurements were done on wood samples in the field, which integrated whole-canopy WUE as well as post-photosynthetic discrimination (Badeck *et al.*, 2005). This suggests that the differences in stomatal conductance among the seedlings were probably cumulative over time and the crown, resulting in a much stronger differentiation among older trees and no detectable relationship with aboveground growth. As far as is known, this is the first time that such a strong QTL for WUE estimated by δ^13^C in a perennial plant has been identified. Similar strong-effect QTLs (21–64% of PEV) have only been detected in recombinant inbred lines on *Arabidopsis thaliana* (Masle *et al.*, 2005) and *Solanum* spp. introgression lines (Xu *et al.*, 2008). For a perennial species, *Q. robur*, a rather strong QTL (21–31% of PEV) for δ^13^C has also been detected with a multiyear QTL analysis (Brendel *et al.*, 2008). In other perennial species, 2–17 QTLs for δ^13^C have been detected, explaining each from 2 to 18% of PEV: *Pinus pinaster* (Brendel *et al.*, 2002), *Gossypium* interspecific cross (Saranga *et al.*, 2004), *Castanea sativa* Mill (Casasoli *et al.*, 2004), *Salix* interspecific population (Rönnberg-Wästljung *et al.*, 2005; Weih *et al.*, 2006), *Populus* interspecific cross (Street *et al.*, 2006; Monclus *et al.*, 2012), and woody fruit crops (*Vitis* spp., Marguerit *et al.*, 2012; *Malus domestica*, Regnard *et al.*, 2009; Segura *et al.*, 2009). In these studies, sample sizes varied from 92 to 331, which, according to simulations by Beavis *et al.* (1994), would suggest that QTL effects were overestimated. In annual crops, genetic control of δ^13^C has been shown to be under the control of several independent loci explaining a small part of PEV, not repeatable across years, and thus subject to large QTL–environment interactions (Rebetzke *et al.* 2008). However, this is not necessarily the case, as shown by Brendel *et al.* (2008) for pedonculate oak, where several QTLs were detectable across years. In the present study, the relevant choice of parents with contrasting behaviour under water deficit (Landes and Corsican genotypes), the large variability present in the F2 population, a potential cumulative effect with ageing for the QTLs, and the size of the population (447 phenotyped trees) allowed the reliable detection of a large-effect QTL.

### Relationship between δ^13^C and growth traits

At the interprovenance level, several studies have analysed the correlation between δ^13^C and growth in maritime pine. Nguyen-Queyrens *et al.* (1998) found a positive phenotypic correlation between tree height and δ^13^C with trees from the French Landes and the Moroccan Tamjoute provenance. On the contrary, Correia *et al.* (2008) found a negative correlation between height and δ^13^C across provenances from the Atlantic coast (France, Spain, and Portugal), as did Corcuera *et al.* (2010) across three Spanish populations and Lamy *et al.* (2011) for French, Spanish, and Moroccan provenances.

At the within-provenance level, Brendel *et al.* (2002) showed a positive phenotypic correlation between ring width and δ^13^C but this correlation appears not significant at the genetic level. Enlarging the literature review to other forest trees evaluated in field after the juvenile age, no general trend could be highlighted. Some authors found negative correlations, such as Xu *et al.* (2000) in *Pinus elliottii* × *Pinus caribaea*, Johnsen *et al.* (1999) in *Picea mariana*, and Lauteri *et al.* (2004) in *C. sativa*, while others showed positive correlations, such as Prasolova *et al.* (2001) and Xu *et al.* (2003) in *Araucaria cunninghamii* or nonsignificant correlations as Ponton *et al.* (2001) in *Quercus petraea*. These results can be first explained by a poor estimation of the genetic correlation due to limited sampling, as δ^13^C measurements are cost and time demanding. This discrepancy also suggests that the direction of this correlation is driven by different physiological mechanisms depending on the species and even ecotype considered. A positive correlation between WUE and growth could suggest that WUE is primarily determined by leaf assimilation, which would impact variability of biomass accumulation. On the other hand, a negative correlation could indicate that WUE is primarily determined by stomatal conductance, having little impact on biomass accumulation.

No significant unfavourable genetic correlations were found between δ^13^C and growth traits. Thus, it appears possible to select maritime pine for δ^13^C without counter selection for growth within the Landes genepool. Moreover, highly significant positive (favourable) correlations were estimated from the dry site (Escource), suggesting that the acclimation to this site resulted in an increased WUE that was genetically linked with an increase of growth.

As all three planting sites are within a small region in the southwest of France, the overall climate is similar and the differences in soil water content are mainly dependent on the soil type. The driest soil at Escource, which contained the highest proportion of sand, also showed relatively low nitrogen and phosphorous contents. The resulting nutrient limitation was therefore probably higher in the dry site due to reduced nitrogen mineralization compared to the other sites (Stanford and Epstein, 1974; Cassman and Munns, 1980). Thus, in addition to variability in WUE among genotypes, variability in nutrient-use efficiency, which has an impact on photosynthetic capacity, could be the cause of the observed positive genetic correlation between growth and δ^13^C. Disentangling the effect of both factors will require further investigation.

In the plantation population, there was no coincidence between the major QTLs for δ^13^C and growth traits. In addition, there was no phenotypic correlation (*R*=–0.04, not significant) between these two traits. Additionally, the test of the homogeneous group at the closest markers from the QTLs did not show the same pattern of significant differences between the homozygous and heterozygous groups (Supplementary Table S8 available at *JXB* online), suggesting no common genetic regulation between growth and δ^13^C. These results therefore suggest that the genetic determinisms of δ^13^C and ring width are independent. The absence of relationships between growth and δ^13^C has also been found in *Populus* interspecific families (Marron *et al.*, 2005; Monclus *et al*., 2005, 2006, 2012; Dillen *et al.*, 2008). The absence of a trade-off between these traits suggests that it should be possible to select maritime pine genotypes combining high growth and high WUE.

### Conclusions

This work presents a comprehensive genetic study over multiple sites and different ages that quantify the genetic relationship between growth and WUE. A large sampling of the maritime pine breeding population (Landes provenance), established in sites contrasting in water availability, showed medium heritability for δ^13^C, an indirect measure of intrinsic WUE. No unfavourable correlation with growth was found in this population. The genetic variability for δ^13^C appeared higher in a Landes × Corsica F2 population and the genetic architecture revealed a strong QTL detected in the same genomic region in two experimental settings.

The expected genetic gain for a selection for δ^13^C in the Landes breeding population was rather low (shift of 0.4‰ if the best 1% of genotypes are selected). However, the Corsican genetic background, which brings a recessive allele improving WUE greatly by decreasing stomatal conductance with only little effect on assimilation rate and growth, could be used in controlled crosses with the Landes genetic background. More generally, it seems interesting to explore the genetic diversity for WUE within the Corsican gene pool for implementation in the maritime pine breeding programme. The usefulness of WUE, estimated by δ^13^C, is under debate in breeding of annual crops for higher yield under drought conditions (Blum, 2009; Chen *et al.*, 2011; Sinclair, 2012), with the main argument that often higher WUE is linked to lower stomatal conductance, which then also decreases carbon assimilation and thus yield; however, for the maritime pine genetic backgrounds studied here, higher WUE was not genetically linked to less growth and there is therefore space for improvement in water use.

The genetic improvement of the Landes breeding population in itself for WUE might not be a major aim. Rather, more research is needed to support the interest of the Corsican genetic background in hybrid combination with the Landes provenance, which is well adapted to the Aquitaine region, especially in terms of frost resistance. To gain more insights into the functional background of observed variability in WUE, an approach of choosing specific genotypes for detailed studies, as has been done for pedonculate oak (Brendel *et al.*, 2008; Roussel *et al*., 2009a, b), might be feasible. The genetic material created within the maritime pine breeding programme (recurrent selection scheme between the Landes and Corsican genepools in the early 1980s and large progeny provenance tests of Corsican ecotypes in the 2010s) provide further research grounds to study the genetics of WUE in this species.

## Supplementary material

Supplementary data are available at *JXB* online.

Supplementary Table S1. Main characteristics of the studied trials and associated annual measurements.

Supplementary Table S2. Soil composition in the three HST sites.

Supplementary Table S3. Main characteristics of the four genetic linkage maps used in this study.

Supplementary Table S4. Pearson’s correlation coefficients between water-use efficiency estimated by δ^13^C for the F2 greenhouse population

Supplementary Table S5. Means, phenotypic coefficients of variation, and additive coefficients of variation for δ^13^C in the HST and mapping populations.

Supplementary Table S6. Pearson’s correlation coefficients and linear correlation slopes between *A*, *g*_s_, *A*/*g*_s_, δ^13^C, and woody biomass for the F2 greenhouse population.

Supplementary Table S7. Significant QTLs for WUE estimated by *A*/*g*_s_ and for each component of the previous ratio for the F2 greenhouse population.

Supplementary Table S8. Effect of the Corsican recessive allele on WUE estimated by δ^13^C and mean ring width in the F2 plantation population.

Supplementary Table S9. Correspondence between linkage groups of the different genetic maps used in this study.

Supplementary Fig. S1. Distribution of breeding values for height at 12 years in the G1 population and the sampled G1 population.

Supplementary Fig. S2. Relationship between stomatal conductance of water vapour and net CO_2_ assimilation rate.

Supplementary Fig. S3. Individual measurements of δ^13^C for the F2 plantation population.

Supplementary Data
