# Supplementary material for: The genetics of water-use efficiency and its relation to growth in maritime pine
Source: J Exp Bot. 2014 Jul 1;65(17):4757–68. doi: 10.1093/jxb/eru226 (PMC4144764; doi:10.1093/jxb/eru226)
Supplement: Supplementary Data [file supp_65_17_4757__index.html]

The genetics of water-use efficiency and its relation to growth in maritime pine — The genetics of water-use efficiency and its relation to growth in maritime pine — Supplementary Data 

# The genetics of water-use efficiency and its relation to growth in maritime pine

## Supplementary Data

Data files

**Files in this Data Supplement:**

- Supplementary Data - Supplementary Data
