# Supplementary material for: The genetics of water-use efficiency and its relation to growth in maritime pine
Source: J Exp Bot. 2014 Jul 1;65(17):4757–68. doi: 10.1093/jxb/eru226 (PMC4144764; doi:10.1093/jxb/eru226)
Supplement: Supplementary Data [file supp_eru226_jexbot116319_file001.pdf]

The genetics of water-use efficiency and its relation to growth in maritime pine

Elisa Marguerit[1,2,3*§], Laurent Bouffier[3,4*], Emilie Chancerel[3,4], Paolo Costa[3,4†], Frédéric Lagane[3,4], Jean-Marc Guehl[5,6], Christophe Plomion[3,4], Oliver Brendel[5,6]

Supporting Information

Supplementary Table S1. Main characteristics of the studied trials and associated annual measurements

Trials		GPS coordinates	Plantation year	Experimental conditions	References	Pedigree	Sample size	Phenotypic data	
								**Water Use Efficiency**	**Growth related traits**
**HST (half-sib families trial)**	**Escource (dry site)**	44°10'N 1°01'W	1996	Field	-	half-sib families	634	**δ^{13}C**: measured on one block of 3 annual rings (2006, 2007 and 2008)	**Ht_12**: height at 12 years<br>**Cir_12**: girth at 12 years<br>**RW_2006-2008**: ring width for years 2006 to 2008
	**Lagnereau (semi-humid site)**	44°30'N 1°01'W					636		
	**Cestas (humid site)**	44°42"N 0°46'W					631		
**mapping populations F2**	**F2-greenhouse**	44°44'N 0°46'W	1995	Pots in greenhouse	(Costa, 1999)	inbred pedigree	200	**δ^{13}C_stem_WW; δ^{13}C_shoot_WW; δ^{13}C_stem_D; δ^{13}C_shoot_D**: δ^{13}C measured in well watered (WW) or dry (D) conditions on stem or lateral shoot<br>**A**: CO_2 assimilation rate<br>**g_s**: leaf conductance	**Biomass**
	**F2-plantation**	44°39'N 0°54'W	1999	Field	-		470	**δ^{13}C**: measured on one block of 3 annual rings (2006, 2007 and 2008)	**RW_2006-2008**: ring width for years 2006 to 2008
**G2**		44°30'N 0°47'W	1982	Field	(Brendel *et al.*, 2002)	outbred pedigree	202	**δ^{13}C**: measured on cellulose extracted on block of 4 rings (1993 to 1996)	**RW_1993-1996**: ring width for years 1993 to 1996

Supplementary Table S2. Soil composition in the three HST sites (each value is a mean of three measurements at depth 0-20cm, 20-40cm and 40-60cm)

	Escource	Lagnereau	Cestas
**Organic matter (%)**	2.10	3.10	3.73
**Nitrogen (%)**	0.05	0.06	0.09
**Ratio C/N**	20.5	27.4	25.0

Supplementary Table S3. Main characteristics of the four genetic linkage maps used in this study

Genetic map ID	Pedigree	Number of genotypes	Molecular markers	Reference for the genetic map	Traits phenotyped	Reference for QTL analysis
**MapF2g**	F2-greenhouse	200	23 AFLPs<br>127 RAPDs<br>32 protein markers	(Costa *et al.*, 2000)	$\delta^{13}C$<br>g_s<br>A/g_s	This study
**MapF2p**	F2-plantation	477	265 SNPs	(Chancerel *et al.*, 2011)	$\delta^{13}C$<br>ring width mean	This study
**Map 1**	A different set of gametophytes than F2-greenhouse	124	436 RAPDs<br>5 SSCPs<br>17 protein markers	(Plomion *et al.*, 1995)<br>(Plomion *et al.*, 1997)	-	-
**Map2♀ and Map2♂**	Three-generation outbred pedigree	186	219 AFLPs	(Chagné *et al.*, 2002)<br>(Pot *et al.*, 2006)	$\delta^{13}C$<br>ring width mean	(Brendel *et al.*, 2002)

Supplementary Table S4. Pearson's correlation coefficients between water use efficiency estimated by $\delta^{13}C$ analysed on needles sampled in well watered conditions (a) on main terminal ($\delta^{13}C$_WW_terminal) (b) on lateral lateral ($\delta^{13}C$_WW_lateral), and in drought conditions (c) on main terminal ($\delta^{13}C$_D_terminal) (d) on lateral lateral ($\delta^{13}C$_D_lateral), for the F2-greenhouse population

R	$\delta^{13}C$_WW_terminal	$\delta^{13}C$_WW_lateral	$\delta^{13}C$_D_terminal	$\delta^{13}C$_D_lateral
$\delta^{13}C$_WW_terminal	1	0.93	0.92	0.88
$\delta^{13}C$_WW_lateral		1	0.89	0.90
$\delta^{13}C$_D_terminal			1	0.85
$\delta^{13}C$_D_lateral				1

Supplementary Table S5. Mean (‰), phenotypic coefficient of variation (CV_P) and additive coefficient of variation (CV_A) for $\delta^{13}C$ in HST (Escource, Lagnereau and Cestas) and mapping populations (F2-greenhouse and F2-plantation).

	Escource	Lagnereau	Cestas	F2-greenhouse	F2-plantation
**Mean**	-25.7	-26.2	-26.4	-28.3%	-26.4%
**CV_P**	1.9%	2.0%	2.0%	3.1%	2.6%
**CV_A**	0.9%	1.3%	1.1%	-	-

Supplementary Table S6. Pearson's correlation coefficients above the diagonal and linear correlation slope below the diagonal, between photosynthetic capacity (A), stomatal conductance (gs), the ratio A/ gs, δ13C and woody biomass including stem, branches without needles and roots, for the F2-greenhouse population.
(* significant at level 5%, ** significant at level 1%)

R	A	g_s	A/g_s	$\delta^{13}C$	Woody biomass
**A**	X	0.42**	-0.18*	-0.19**	-0.07
**g_s**	0.02	X	-0.88**	-0.53**	-0.27**
**A/g_s**	-5.3	-407	X	0.48**	0.27**
**$\delta^{13}C$**	-0.4	-16.5	0.03	X	0.07
**Woody biomass**	-0.02	-1.2	0.002	0.02	X

Supplementary Table S7. Significant quantitative trait loci (QTL) of water use efficiency estimated by the ratio of photosynthesis activity (A) and leaf stomatal conductance (gs), and for each component of the previous ratio, for the F2-greenhouse population phenotyped in well watered conditions (198 plantlets).

Abbreviations: N number of trees phenotyped, *LG* linkage group, p_G level of significance at the genome level, *L* position of QTL on the LG in cM estimated from original data-set, L_{BS} position of QTL as calculated from bootstrap analysis ± standard deviation, [-] is the confidence interval for p =0.05, *PEV* percentage of explained variance, *s* allelic substitution effect, effects marked with * are significantly different from zero.

Trait	LG	LOD	p_G	L cM	L_{BS} cM	[-]at 0.05%	PEV %	s
**A/g_s**	12	2.8	0.04	34.1	40.0 ± 26.3	0-91.5	6.5	0.033*
**A**	-	-	-	-	-	-	-	-
**g_s**	12	3.5	0.0004	32.8	39.3 ± 24.4	0-87.2	7.2	-15.1*

Supplementary Table S8. Effect of the Corsican recessive allele on water use efficiency estimated by $\delta^{13}C$ and mean ring width in the F2-plantation population. SD standard deviation, N number of trees in each class, * from Chancerel et al. (2013)

Molecular markers flanking the QTL on linkage group 12*	Class	N	$\delta^{13}C$		Ring width	
			Mean $\delta^{13}C$ ± SD	Homogeneous group at 1% threshold (Tukey test)	Mean ring width ± SD	Homogeneous group at 1% threshold (Tukey test)
**SNPnew50**	Landes allele homozygous	116	-26.7 ± 0.4	A	7.9 ± 1.6	A
	Corsican allele homozygous	102	-25.6 ± 0.7	B	7.3 ± 1.5	A
	Heterozygous	206	-26.6 ± 0.4	A	7.8 ± 1.6	A
**SNPnew89**	Landes allele homozygous	106	-26.7 ± 0.4	A	8.0 ± 1.6	A
	Corsican allele homozygous	91	-25.6 ± 0.7	B	7.3 ± 1.6	B
	Heterozygous	215	-26.6 ± 0.5	A	7.8 ± 1.4	AB

Supplementary Table S9. Correspondence between linkage groups of the different genetic maps used in this study

Linkage groups from Plomion et al. 1995 and Costa et al. 2000	Linkage groups from Chagné et al. 2004	Linkage groups from Chancerel et al. 2011
9	1	1
7	2	2
3	3	3
8	4	4
2	5	5
1	6	6
4	7	7
10	8	8
11	9	9
5	10	10
12	12	11
6	11	12

Supplementary Figure S1. Distribution of breeding values for height at 12 years (Ht_12) in the G1 population (black) and for G1 sampled population (grey)

Supplementary Figure S2. Relationship between stomatal conductance to water vapor (g_s) and net CO_2 assimilation rate (A_{max}) at saturating light, 200ppm CO_2 and well-watered conditions (black dots); the black line represents a distance weighted least squares trend line of gas exchange data, including also measurement during a developing drought stress (these data were not analyzed in this article and therefore the raw datapoints are not shown). The dashed line represents the geometric mean square correlation.

Supplementary Figure S3. Representation of individual measurements of $\delta^{13}C$ for the F2-plantation (the gird is 4 meters between rows represented here along the y-axis and 2 meters between positions represented here along the x-axis). No spatial pattern was detected, suggesting that no micro-environmental effect may have biased the data.

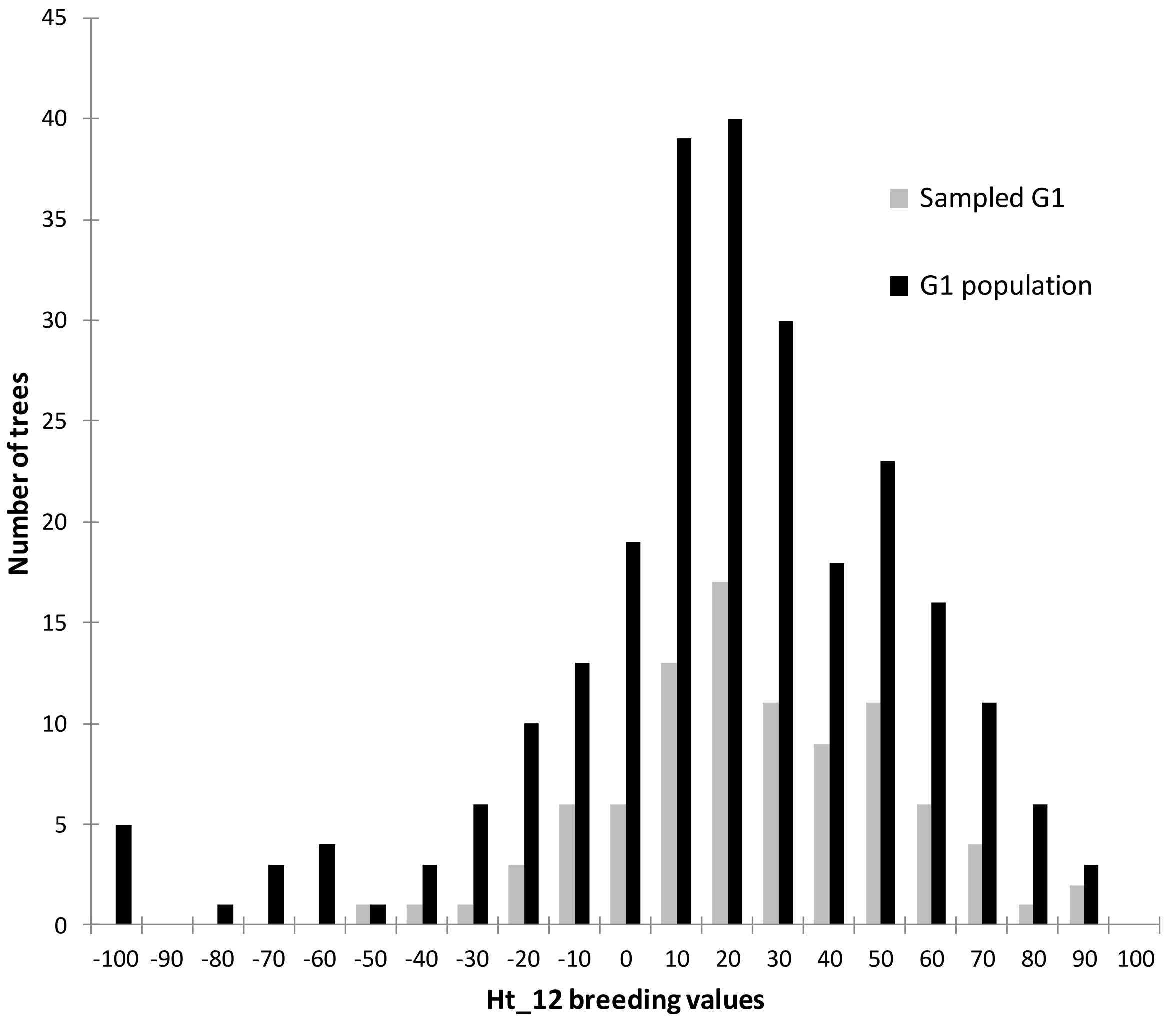

Supplemental Figure S1

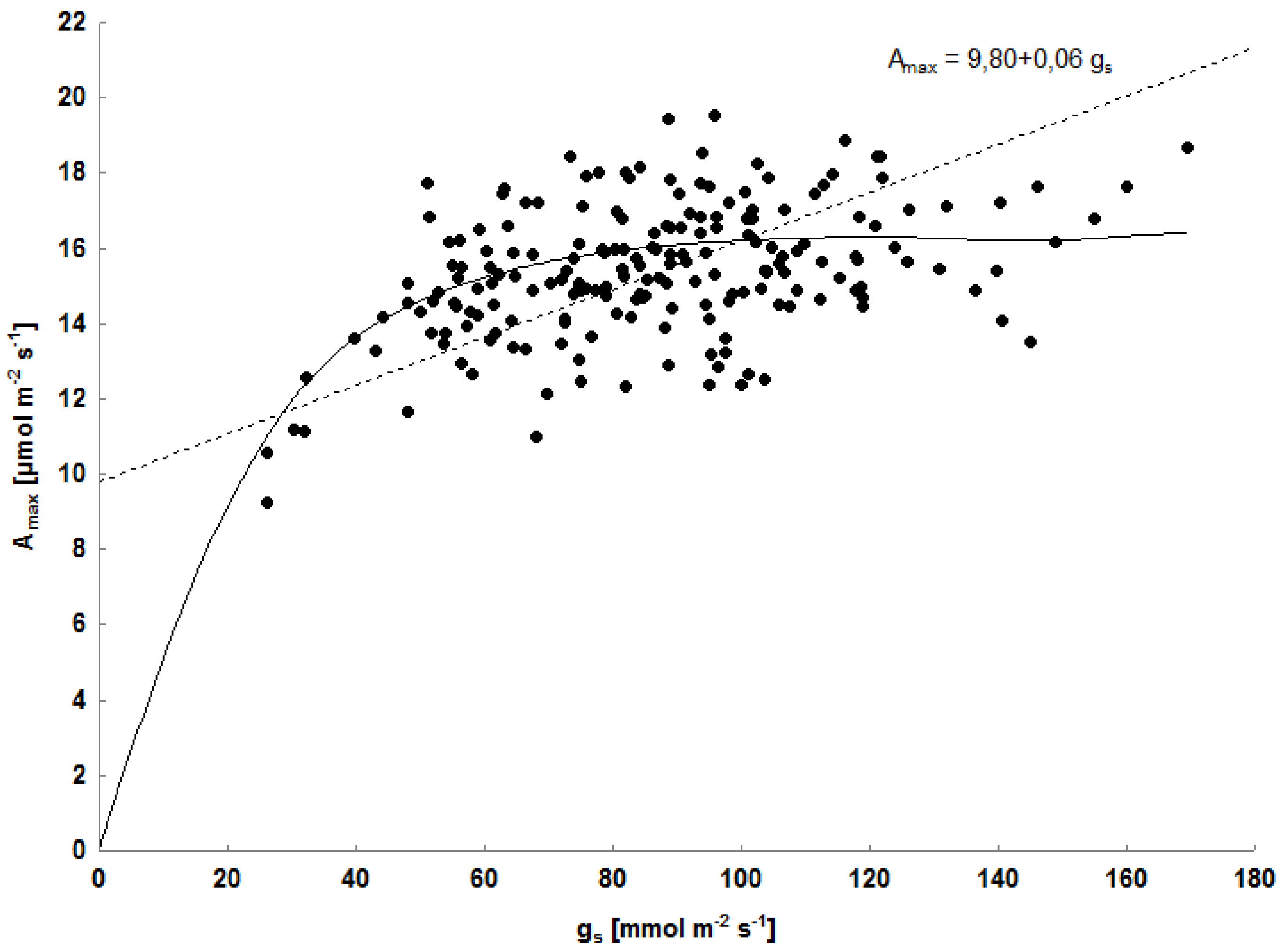

Supplemental Figure S2

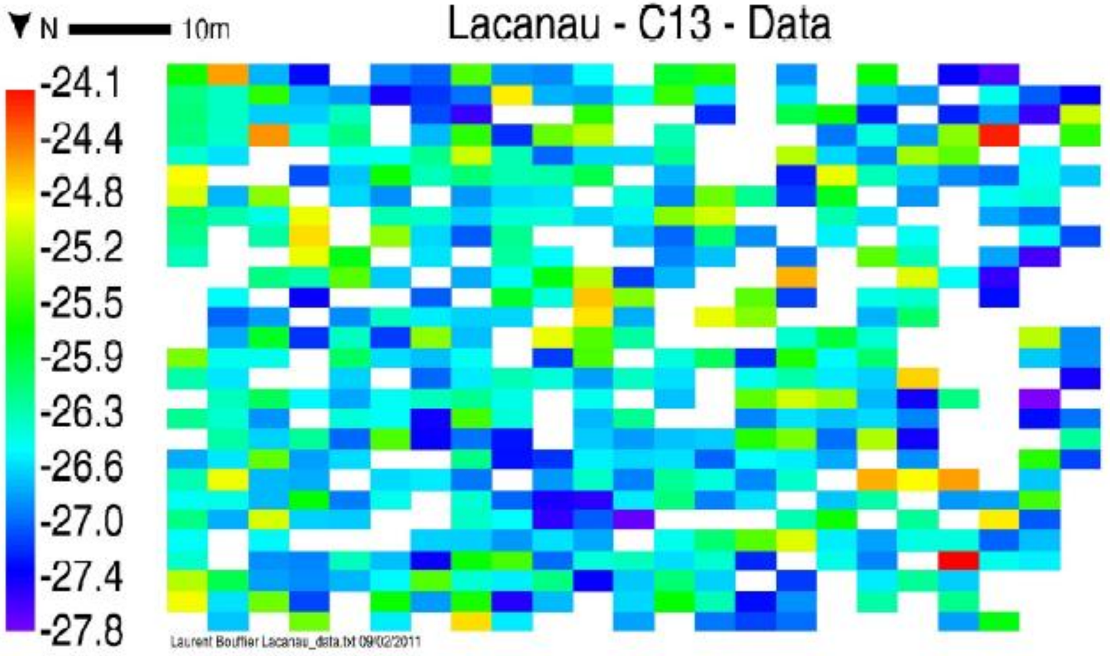

Supplemental Figure S3
